# Supplementary material for: A case against default effect sizes in sport and exercise science
Source: PeerJ. 2020 Nov 3;8:e10314. doi: 10.7717/peerj.10314 (PMC7646309; doi:10.7717/peerj.10314)
Supplement: Supplemental Information 2 [file peerj-08-10314-s002.pdf]

How to Calculate and Report Effect Sizes

Aaron R. Caldwell

2020-08-17

Introduction

In our manuscript, we argued that sport and exercise scientists should avoid the temptation of reporting a "default" effect size. Instead, we suggested that the effect size be chosen on a case-by-case basis and reported in a way that facilitates the appropriate communication of study results. With that said, we realize many sport scientists may not know how to calculate these effect sizes in a efficient manner. Therefore, we have written this document to show how **R** [R Core Team, 2019] can be used for these calculations.

Loading the Right Tools

None of the following packages are 'necessary', and all of these analyses can be performed in 'base' `R`. But, I use these packages to make the analysis pipeline simpler and a little 'cleaner' too. In total, I need the `tidyverse` [Wickham et al., 2019], `boot` [Canty and Ripley, 2019], `broom` [Robinson and Hayes, 2019], and `MASS` [Venables and Ripley, 2002] packages. You will likely not need `MASS` or `broom` for your own analyses, but it is necessary for this document in order to create a simulated dataset (`MASS`) and make the tables in the document (`broom`).

```
#LOAD PACKAGES
library(tidyverse)
library(boot)
library(MASS)
library(broom)
```

Example 1: $\dot{V}O_2$ Data

Generate Some Data

Okay, now I can generate data similar to what we used in the manuscipt. First, let us create an "Interpretatable Raw Differences" example. In this example, we have a pre-to-post design of athletes where we are measuring $\dot{V}O_2$ (L·min^{-1}). Further, we assume we are sampling from a population with a standard deviation of 0.2, pre-post correlation of 0.9, and a mean increase of 0.3 (L·min^{-1}).

```
set.seed(20200303)
var_raw = .2^2
```

```
cor_raw = matrix(c(1,.8,.8,1),nrow=2)
Sigma_raw = cor_raw*var_raw
df_raw = mvrnorm(
  n = 10,
  Sigma = Sigma_raw,
  mu = c(3.9, 4.1)
  ) %>%
  as.data.frame() %>%
  rename(pre = V1,
         post = V2)

#Add a change score column
df_raw = df_raw %>% mutate(pre = round(pre,2),
                           post = round(post,2)) %>%
  mutate(diff = post-pre)
```

Table 1: Raw Differences Data

pre	post	diff
3.85	4.15	0.30
3.75	4.11	0.36
4.03	4.14	0.11
3.95	4.08	0.13
4.22	4.40	0.18
4.20	4.11	-0.09
3.74	3.99	0.25
4.13	4.17	0.04
3.98	4.05	0.07
4.05	4.20	0.15

The Simple Analysis

Now that we have our data, we can perform a simple *t*-test on the difference scores.

```
t_raw = t.test(df_raw$diff)
knitr::kable(tidy(t_raw),caption="Raw Differences t-test",
             digits = 4)
```

Table 2: Raw Differences t-test

estimate	statistic	p.value	parameter	conf.low	conf.high	method	alternative
0.15	3.6075	0.0057	9	0.0559	0.2441	One Sample t-test	two.sided

Okay, so we now have our *t*-statistics, *p*-values, mean differences, and confidence intervals. Pretty painless to get this far, but maybe we want (or need) to get the standardized mean difference (SMD) and common langauge effect sizes (CLES).

Let's Bootstrap

While it is easy to calculate the SMD or CLES in R, it is quite another thing to calculate their confidence intervals (CI). Unlike the mean difference, the formulae for the CI of the SMD are convoluted (see Hedges [1981]). Thus, here, we rely on the bootstrap to generate CIs.[1]

[1] There are some formula for calculating the standard error, and therefore confidence intervals, of SMDs. Many of these calculations are contained in a review by Nakagawa and Cuthill [2007]. However, the required formula is dependent on the SMD being chosen. For example, the SE calculation differs from Cohen's d and Hedges' g, despite the later being a corrected version of the former. To avoid potential miscalculations, we strongly advocate for the use of bootstrapping methods.

First, we must create a function to calculate all the SMD statistics we would like to obtain.

```
# function to obtain R-Squared from the data
SMD <- function(data, indices) {
  d <- data[indices,] # allows boot to select sample
  sd_pre = sd(d$pre, na.rm = TRUE)
  sd_post = sd(d$post, na.rm = TRUE)
  sd_av = (sd_pre+sd_post)/2
  m_diff = mean(d$diff, na.rm = TRUE)
  sd_diff = sd(d$diff, na.rm = TRUE)
  cor_prepost = cor(d$pre,d$post,
                    method = "pearson")
  dz = m_diff/sd_diff
  dav = m_diff/sd_av
  glass = m_diff/sd_pre
  drm = m_diff/sqrt((sd_pre^2+sd_post^2)-(2*cor_prepost*sd_pre*sd_post))*sqrt(2*(1-cor_prepost))
  CLES = pnorm(dz)
  result = c(dz,drm,dav,glass,CLES)

}
```

Second, we can start bootstrapping these statistics. To do so, we select each value based on their index (1-5) from the function above. We must also specify the *type* of bootstrap CI. In this case, I have decided to report the bias-corrected and accelerated (BCa) intervals as they will likely provide our most accurate estimate.

```
raw_boot = boot(df_raw, SMD, R = 2000)
#Extract the values
dz_raw = boot.ci(raw_boot, type="bca", index=1)
drm_raw = boot.ci(raw_boot, type="bca", index=2)
dav_raw = boot.ci(raw_boot, type="bca", index=3)
glass_raw = boot.ci(raw_boot, type="bca", index=4)
CLES_raw = boot.ci(raw_boot, type="bca", index=5)
```

From these statistics, we can extract the effect size estimate by calling on the `$t0` part of the saved object.

For example:

```
dz_raw$t0
```

```
## [1] 1.140795
```

And the upper limit and lower limit of the 95% CI can also be found in the `dz_raw$bca` object. The last and second-to-last numbers represent the upper limit and lower limit of the 95% CI.

```
dz_raw$bca
```

```
##      conf
## [1,] 0.95 3.44 1806.43 0.1956553 1.991893
```

Finally, we can put all of the SMD calculations in a summary table.

Table 3: Effect Sizes for ‘Raw’ Differences Dataset

Effect Size	Estimate	Lower Limit	Upper Limit
d(z)	1.1408	0.1957	1.9919
d(rm)	0.9688	0.3886	1.5554
d(av)	1.0689	0.4172	1.8032
Glass’s Delta	0.8771	0.2360	1.2969
CLES	0.8730	0.5714	0.9753

Example 2: VAS Data

Generate Some Data

Now, we want to create an “Uninterpretable Raw Differences” dataset. This time we are creating a dummy dataset of VAS scores with 15 ‘point’ reduction and correlation of 0.7. Since sensation data are typically distributed log-normal, we will also work with a latent normal distribution.

```
set.seed(20201113)

v = 13^2 # observed variance (log-normal)
m = c(50,35) # observed means (log-normal)
phi = sqrt(v + m^2)
sd_stan = sqrt(log(phi^2/m^2)) # latent SD (normal)
mu_stan = log(m^2/phi) # latent mean (normal)
cor_stan = matrix(c(1,.7,.7,1),nrow = 2)
Sigma_stan = (sd_stan %*% t(sd_stan)) * cor_stan

# Generate latent ratings
df_stan = mvrnorm(
  n = 10,
  Sigma = Sigma_stan,
  mu = mu_stan
) %>%
  as.data.frame() %>%
  rename(pre = V1,
         post = V2)

#Add a change score column
# Latent perceptions
df_stan = df_stan %>% mutate(pre = round(pre,2),
                             post = round(post,2)) %>%
  mutate(diff = post - pre)

# Observed perceptions (log-normal)
df_stan_obs = exp(df_stan[,1:2]) %>%
  mutate(diff = post - pre)
```

Table 4: Log-transformed Difference Data

pre	post	diff
3.52	2.93	-0.59
3.83	3.34	-0.49
3.50	3.53	0.03
4.14	3.60	-0.54
3.56	2.97	-0.59
3.99	3.23	-0.76
3.71	2.83	-0.88
4.01	3.49	-0.52
3.74	3.38	-0.36
4.09	3.43	-0.66

The Simple Analysis

Now that we have our data, we can perform a simple *t*-test on the difference scores, both latent and observed.

```
t_stan_latent = t.test(df_stan$diff)
knitr::kable(tidy(t_stan_latent),caption="Log-transformed Differences t-test",
             digits = 4)
```

Table 5: Log-transformed Differences t-test

estimate	statistic	p.value	parameter	conf.low	conf.high	method	alternative
-0.536	-6.8978	1e-04	9	-0.7118	-0.3602	One Sample t-test	two.sided

```
t_stan_obs = t.test(df_stan_obs$diff)
knitr::kable(tidy(t_stan_obs),caption="Observed Differences t-test",
             digits = 4)
```

Table 6: Observed Differences t-test

estimate	statistic	p.value	parameter	conf.low	conf.high	method	alternative
-19.0403	-6.6056	1e-04	9	-25.5608	-12.5197	One Sample t-test	two.sided

Since the transformation is nonlinear, the expected difference in the observed data is not a simple transformation of the expected difference of the log-transformed data.

Let's Bootstrap

Using the same methods and functions as before, we will use the bootstrap to calculate CIs for each of the SMDs and CLES.

```
#Repeat for the log-transformed dataset
stan_boot = boot(df_stan, SMD, R = 2000)
dz_stan = boot.ci(stan_boot, type="bca", index=1)
drm_stan = boot.ci(stan_boot, type="bca", index=2)
dav_stan = boot.ci(stan_boot, type="bca", index=3)
glass_stan = boot.ci(stan_boot, type="bca", index=4)
CLES_stan = boot.ci(stan_boot, type="bca", index=5)

## Warning in norm.inter(t, adj.alpha): extreme order statistics used as endpoints

#Repeat for the observed dataset
stan_obs_boot = boot(df_stan_obs, SMD, R = 2000)
```

```
dz_stan_obs = boot.ci(stan_obs_boot, type="bca", index=1)
drm_stan_obs = boot.ci(stan_obs_boot, type="bca", index=2)
dav_stan_obs = boot.ci(stan_obs_boot, type="bca", index=3)
glass_stan_obs = boot.ci(stan_obs_boot, type="bca", index=4)
CLES_stan_obs = boot.ci(stan_obs_boot, type="bca", index=5)
```

From these statistics, we can extract the effect size estimate by calling on the `$t0` part of the saved object.

For example, if we look at the latent SMD:

```
dz_stan$t0
```

```
## [1] -2.181274
```

And the upper limit and lower limit of the 95% CI can also be found in the `dz_stan$bca` object. The last and second-to-last numbers represent the upper limit and lower limit of the 95% CI.

```
dz_stan$bca
```

```
##      conf
## [1,] 0.95 253.93 1999.86 -4.571156 -0.8416896
```

Finally, we can put all of the SMD calculations in a summary table. Note, the effect sizes in the 2nd table are negative; that is because there was a decrease from pre-to-post, and the CLES should be reported as $1 - CLES$ from the table.

Table 7: Effect Sizes for Log-transformed Differences

Effect Size	Estimate	Lower Limit	Upper Limit
d(z)	-2.1813	-4.5712	-0.8417
d(rm)	-2.0781	-2.7957	-1.3713
d(av)	-2.0925	-2.7204	-1.2903
Glass's Delta	-2.2349	-3.1933	-1.2703
CLES	0.0146	0.0000	0.2070

Table 8: Effect Sizes for Observed Differences

Effect Size	Estimate	Lower Limit	Upper Limit
d(z)	-2.0889	-3.8253	-0.8166
d(rm)	-1.9477	-3.0301	-1.2459
d(av)	-2.1265	-2.9616	-1.3368
Glass's Delta	-1.7276	-2.3951	-1.0051

Effect Size	Estimate	Lower Limit	Upper Limit
CLES	0.0184	0.0001	0.2142

References

Angelo Canty and B. D. Ripley. *boot: Bootstrap R (S-Plus) Functions*, 2019. R package version 1.3-24.

Larry V. Hedges. Distribution theory for glass's estimator of effect size and related estimators. *Journal of Educational Statistics*, 6 (2):107–128, June 1981. DOI: 10.3102/10769986006002107. URL `https://doi.org/10.3102/10769986006002107`.

Shinichi Nakagawa and Innes C. Cuthill. Effect size, confidence interval and statistical significance: a practical guide for biologists. *Biological Reviews*, 82(4):591–605, Nov 2007. DOI: 10.1111/j.1469-185x.2007.00027.x. URL `http://dx.doi.org/10.1111/j.1469-185X.2007.00027.x`.

R Core Team. *R: A Language and Environment for Statistical Computing*. R Foundation for Statistical Computing, Vienna, Austria, 2019. URL `https://www.R-project.org/`.

David Robinson and Alex Hayes. *broom: Convert Statistical Analysis Objects into Tidy Tibbles*, 2019. URL `https://CRAN.R-project.org/package=broom`. R package version 0.5.3.

W. N. Venables and B. D. Ripley. *Modern Applied Statistics with S*. Springer, New York, fourth edition, 2002. URL `http://www.stats.ox.ac.uk/pub/MASS4`. ISBN 0-387-95457-0.

Hadley Wickham, Mara Averick, Jennifer Bryan, Winston Chang, Lucy D'Agostino McGowan, Romain François, Garrett Grolemund, Alex Hayes, Lionel Henry, Jim Hester, Max Kuhn, Thomas Lin Pedersen, Evan Miller, Stephan Milton Bache, Kirill Müller, Jeroen Ooms, David Robinson, Dana Paige Seidel, Vitalie Spinu, Kohske Takahashi, Davis Vaughan, Claus Wilke, Kara Woo, and Hiroaki Yutani. Welcome to the tidyverse. *Journal of Open Source Software*, 4(43):1686, 2019. DOI: 10.21105/joss.01686.
