# Supplementary figures and images for: A case against default effect sizes in sport and exercise science
Source: PeerJ. 2020 Nov 3;8:e10314. doi: 10.7717/peerj.10314 (PMC7646309; doi:10.7717/peerj.10314)

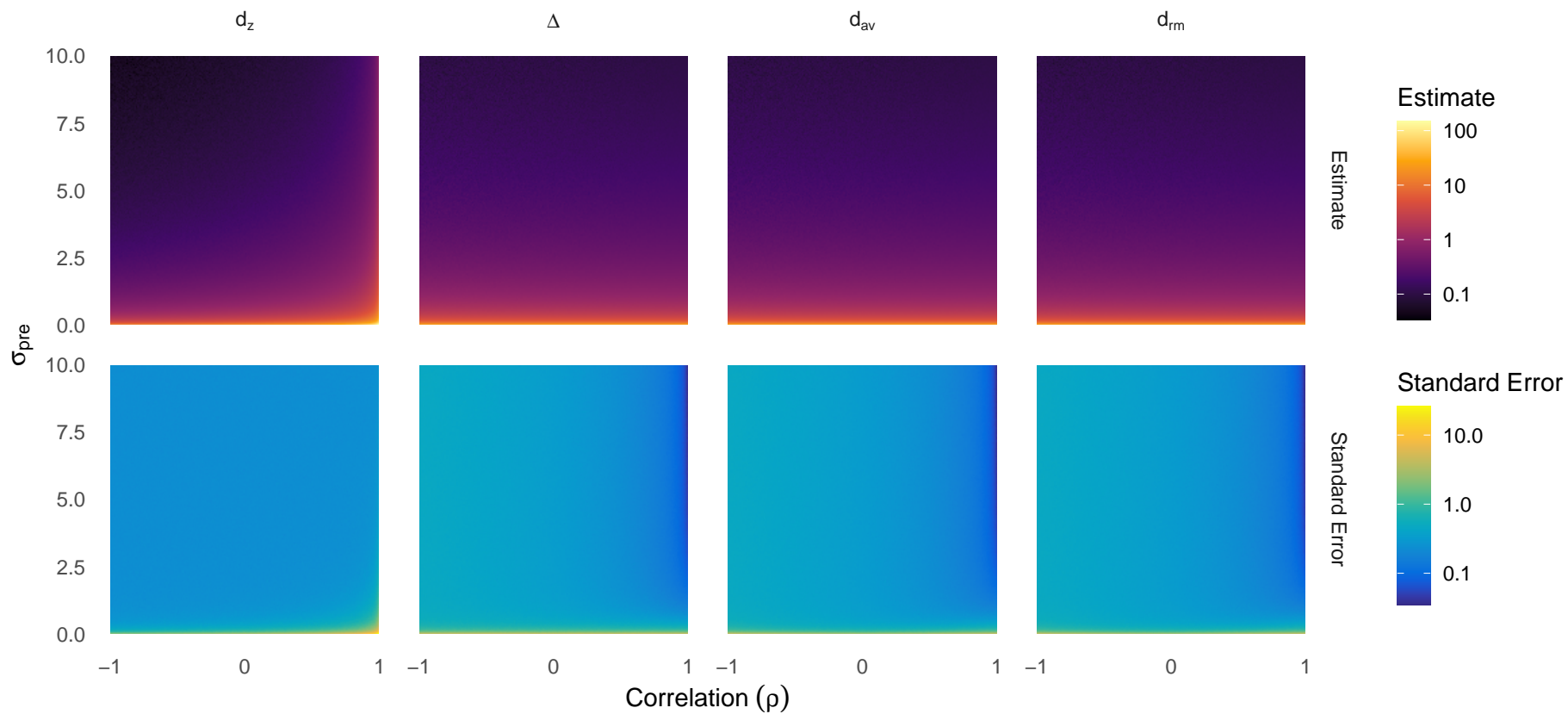

Supplement: Supplemental Information 3 — Simulated standardized mean differences for a range of pre-post correlations and pre-intervention standard deviations. Standardized mean differences (SMD) were simulated for a pre-post design study with 20 participants to depict the different properties of the different SMDs. To complement the scenarios Dankel and Leonneke depict, we simulated 1,000 studies for a range of pre-post correlations (rho) and pre-intervention standard deviations (sigma-pre). From this figure, one can clearly see that magnitude-based SMDs have similar estimates across the range of pre-post correlations and only vary as a function of sigma-pre, whereas signal-to-noise SMDs are a function of both sigma-pre and rho. [file peerj-08-10314-s003.pdf]

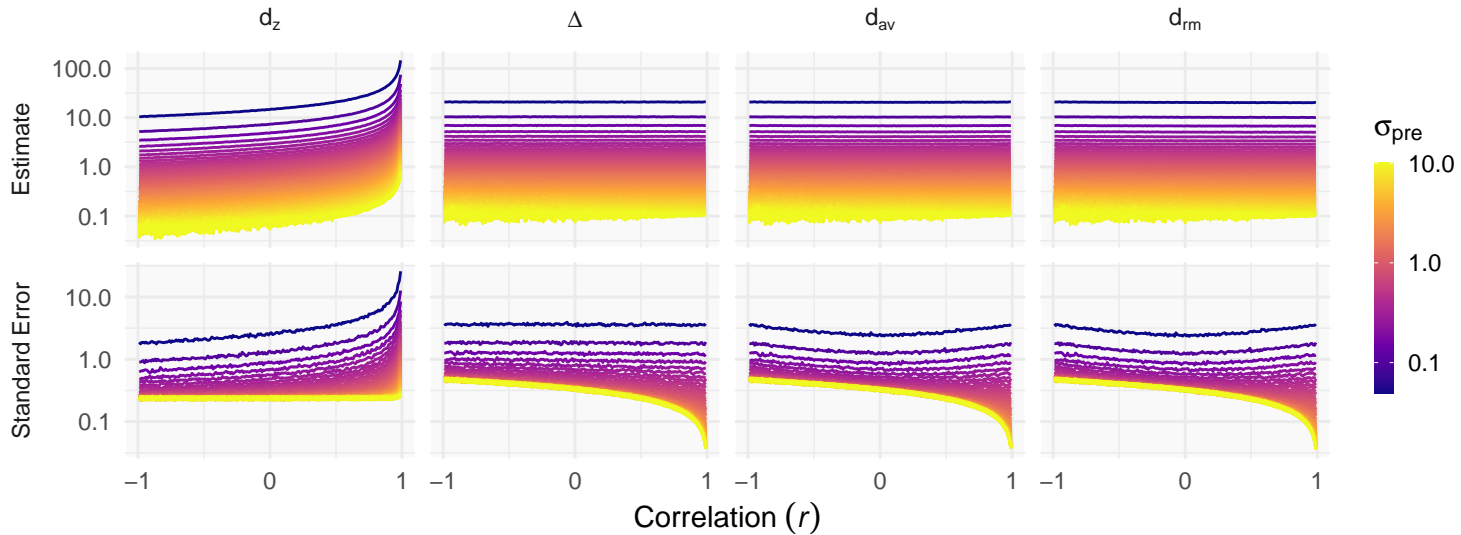

Supplement: Supplemental Information 4 — Simulated standardized mean differences for a range of pre-post correlations and pre-intervention standard deviations. Standardized mean differences (SMD) were simulated for a pre-post design study with 20 participants to depict the different properties of the different SMDs. To complement the scenarios Dankel and Leonneke depict, we simulated 1,000 studies for a range of pre-post correlations (rho) and pre-intervention standard deviations (sigma-pre). From this figure, one can clearly see that magnitude-based SMDs have similar estimates across the range of pre-post correlations and only vary as a function of sigma-pre, whereas signal-to-noise SMDs are a function of both sigma-pre and rho. [file peerj-08-10314-s004.pdf]
